# Cell Cycle-Dependent Expression of Bk Channels in Human Mesenchymal Endometrial Stem Cells

**DOI:** 10.1038/s41598-019-41096-2

**Published:** 2019-03-14

**Authors:** Vladislav I. Chubinskiy-Nadezhdin, Anastasia V. Sudarikova, Mariia A. Shilina, Valeria Y. Vasileva, Tatiana M. Grinchuk, Olga G. Lyublinskaya, Nikolai N. Nikolsky, Yuri A. Negulyaev

**Affiliations:** 1 Institute of Cytology RAS, 194064 Tikhoretsky Ave. 4, St. Petersburg, Russia; 2 Department of Medical Physics, Peter the Great St. Petersburg Polytechnic University, 29, Polytechnicheskaya st., 195251 St. Petersburg, Russia

## Abstract

The study of ion channels in stem cells provides important information about their role in stem cell fate. Previously we have identified the activity of calcium-activated potassium channels of big conductance (BK channels) in human endometrium-derived mesenchymal stem cells (eMSCs). BK channels could have significant impact into signaling processes by modulating membrane potential. The membrane potential and ionic permeability dynamically changes during cycle transitions. Here, we aimed at verification of the role of BK channels as potassium transporting pathway regulating cell cycle passageway of eMSCs. The functional expression of native BK channels was confirmed by patch-clamp and immunocytochemistry. In non-synchronized cells immunofluorescent analysis revealed BK-positive and BK-negative stained eMSCs. Using cell synchronization, we found that the presence of BK channels in plasma membrane was cell cycle-dependent and significantly decreased in G2M phase. However, the study of cell cycle progression in presence of selective BK channel inhibitors showed no effect of pore blockers on cycle transitions. Thus, BK channel-mediated K^+^ transport is not critical for the fundamental mechanism of passageway through cell cycle of eMSCs. At the same time, the dynamics of the presence of BK channels on plasma membrane of eMSCs can be a novel indicator of cellular proliferation.

## Introduction

Ion channels play an important role in numerous cellular reactions in living cells. In stem cells, native ion channels participate in various processes including differentiation, proliferation, cell migration, lineage switching, receptor-induced signaling and other. The expression pattern of ion channels in stem cells significantly varies among different species and sources^1^. Human adult mesenchymal stem cells derived from desquamated endometrium (eMSCs) are promising candidates for use in cell-based therapies due to their availability and non-invasive isolation protocols^2–4^. To date, little is known about the functional expression and the role of ion channels in eMSCs. At the same time, identification and revealing of functional interplay of ion channels in eMSCs might be important in development of new strategies aimed at control of the behavior of particular stem cell line in course of regenerative therapies. Previously, using single channel patch-clamp technique, we have identified several types of native ion channels and revealed their interplay in the plasma membrane of eMSCs. Particularly, the Ca^2+^ -mediated coupling was shown between the activity of Ca^2+^ -dependent potassium ion channels of big conductance (BK, KCa1.1) and mechanosensitive channels^5^. Moreover, our experiments have showed that BK channels are functionally expressed at high level in the plasma membrane; however, the particular role of BK channels in eMSCs remains to be elucidated. Importantly, due to high expression level, BK channels could significantly contribute to different signaling processes in eMSCs via setting and controlling the membrane potential. It is widely recognized, that ionic permeability and membrane potential significantly changes during cell cycle^6^. To date, functional interplay between BK channels, cell cycle progression and proliferation of stem cells or other cell types remain rather controversial^7,8^. Here, we aimed at verification of the putative impact of BK channels as potassium transporting pathway regulating cell cycle passageway of human eMSCs.

## Results

### Patch-clamp and immunofluorescent analysis revealed the expression of BK channels in eMSCs

**Figure 1:**
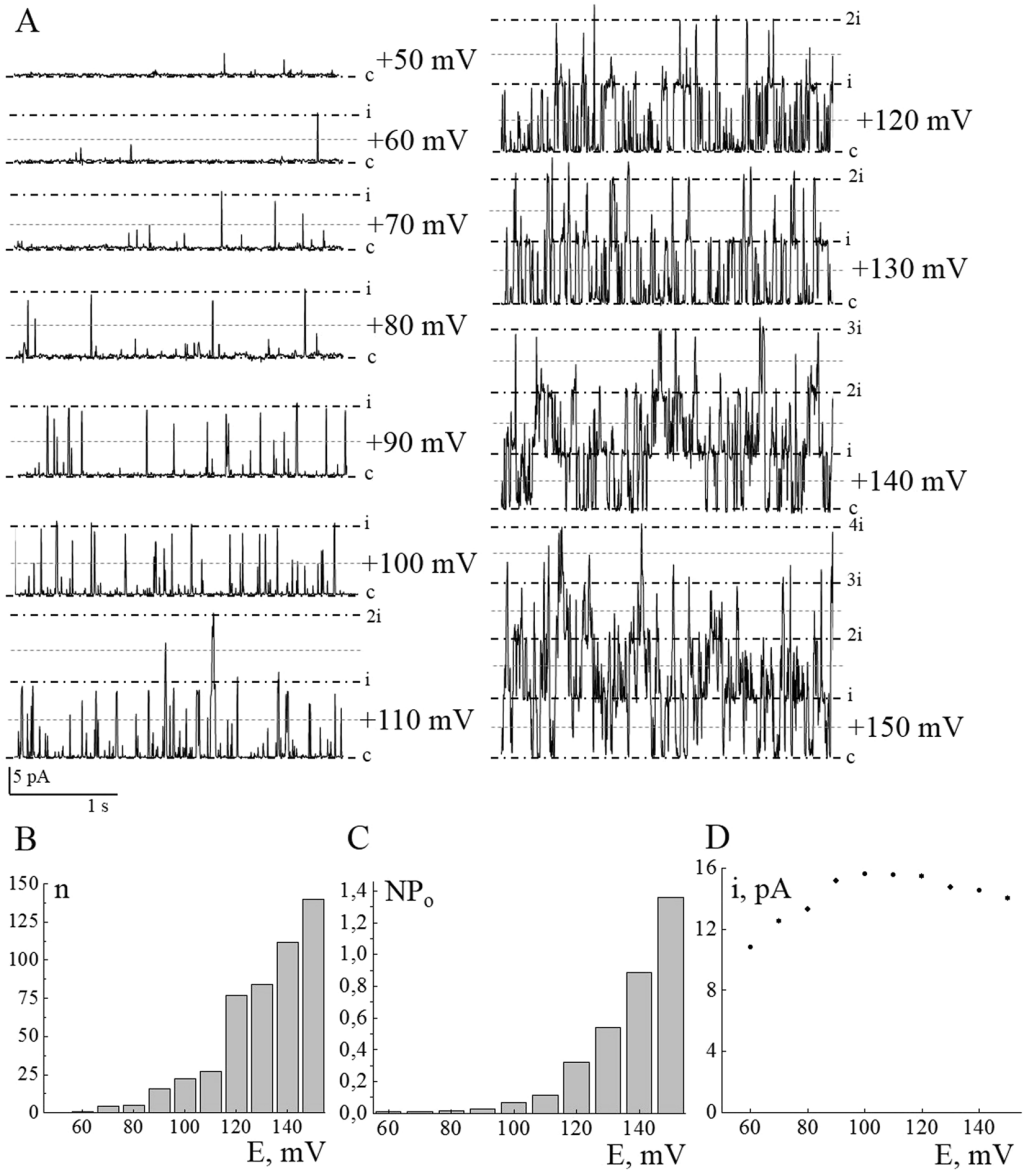
Electrophysiological properties of BK channels in plasma membrane of human eMSC cells. Typical activity of BK channels (**A**) at different holding membrane potentials (indicated near current traces) in cell-attached configuration of patch-clamp technique. Closed state is denoted by (c), open state by (ni), where n is a number of active channels in the patch. The level of 50% open state current of BK channels is marked by blue dotted line. (**B**,**C**) – voltage dependence of BK channel activity: (**B**) the number of channel openings (n) increased with the rise of membrane potential level. The channel was considered as open when current reached 50% of open state. (**C**) NPo of BK channels at different holding potentials. (**D**) Current saturation of BK channels. Single-channel amplitude decreases on membrane potentials higher than +100 mV.

**Figure 2:**
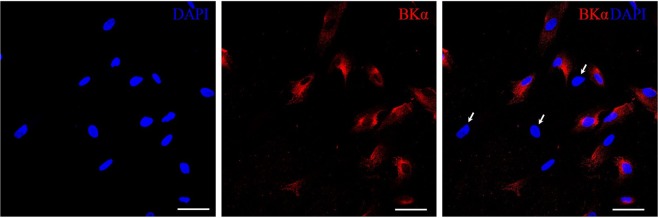
Expression of BK channels in exponentially growing cell culture of eMSCs. Fluorescent labeling of BK channel (red signal, Cy3) and the nuclei (blue signal, DAPI) revealed BK-positive and BK-negative eMSCs (indicated by white arrows). Scale bar 50 µm.

In our study, to confirm the presence of native BK channels in the plasma membrane of eMSCs, patch clamp experiments were performed. The typical activity of BK channels in cell-attached configuration on different holding membrane potentials is shown on Fig. 1A. A number of channel openings and NPo increases in potential-dependent manner (Fig. 1B,C) that is characteristical fingerprint of BK-mediated currents^9^, as well as current saturation (Fig. 1D) at membrane potentials higher than +100 mV^10^. The biophysical characteristics (single channel conductance and reversal potential) of the channels were similar to those recorded previously^5^. Immunofluorescent staining of BK channels with specific antibodies against pore-forming alpha subunit confirmed the expression of BK channels in the plasma membrane of eMSCs (Fig. 2). Importantly, immunofluorescent analysis allowed to detect, that a fraction of cells in exponentially growing eMSC population are not stained with the antibodies (BK-negative cells, Fig. 2). The presence of BK-negative and BK-positive cells could potentially be explained by several factors, including heterogeneity of eMSCs, their differentiation status or the presence of apoptotic cells in culture. To test these possibilities, we confirmed the stemness of eMSCs by immunophenotyping (see Material and Methods and Fig. S2). Our analysis did not reveal differentiated cells in the cell culture and demonstrated the homogeneity of cell population. Furthermore, staining for apoptotic marker Caspase 3/7 demonstrated extremely low basal level of apoptosis in eMSCs culture (Fig. S3), and thus heterogeneity in BK channel expression could not be associated with the cell viability. Instead, we proposed that the difference in BK staining could potentially be explained by cell cycle status of the eMSCs. The changes in membrane permeability as well as the role of different ion channels during cell cycle were reported in numerous studies and reviews forming “membrane potential hypothesis” of cycle progression^6,7^.

### The presence of BK channels in eMSCs is cell cycle-dependent

**Figure 3:**
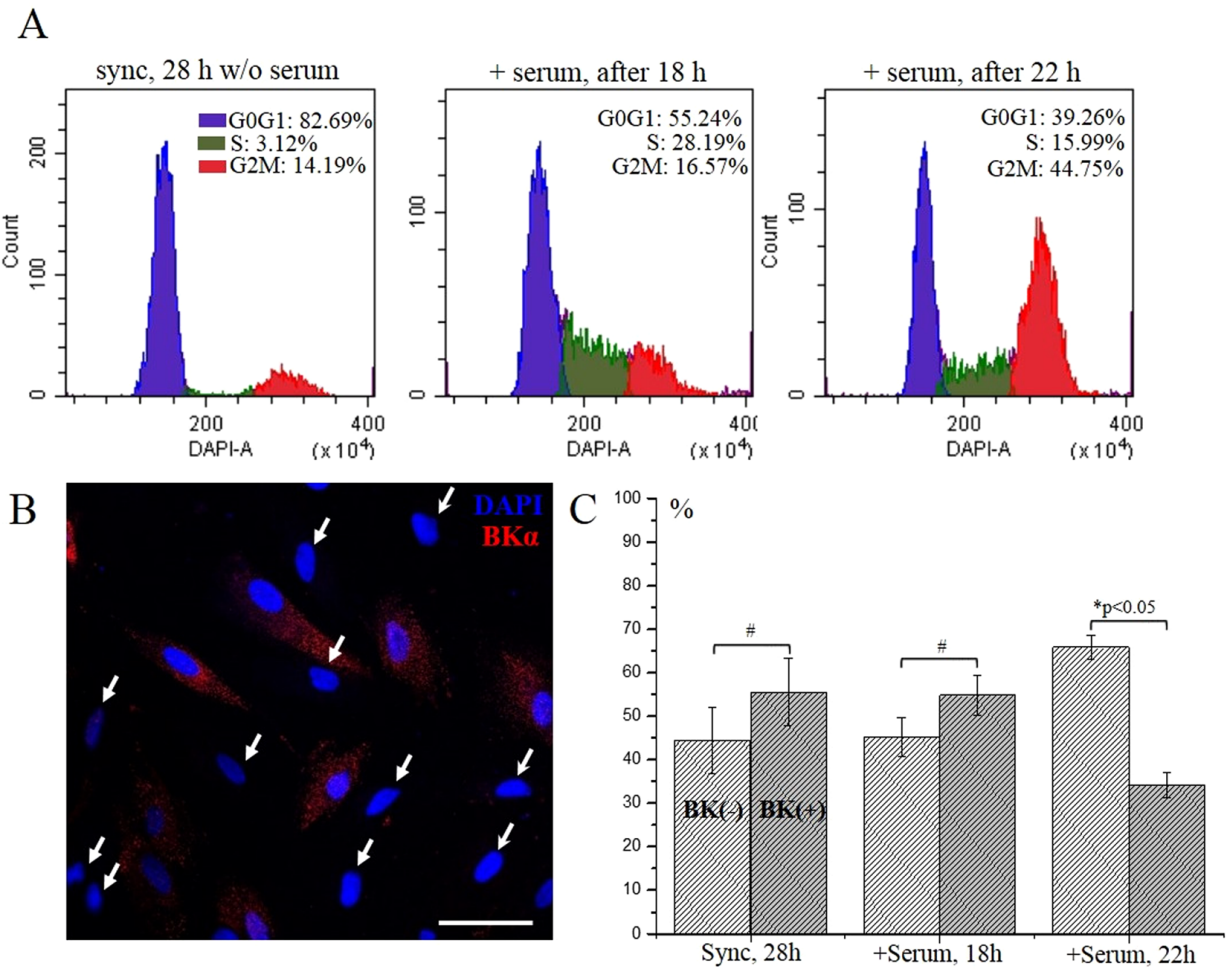
The presence of BK channels on plasma membrane of eMSCs dynamically changes in course of cell cycle progression. (**A**) Cell cycle distribution of synchronized eMSCs after induction of cell proliferation. Shown are flow cytometry histograms of synchronized cells at 18 h and 22 h after serum addition. (**B**) – typical BK staining pattern of eMSCs in G2M phase. BK-negative or BK(−) cells are marked by the arrows. Scale bar 50 µm. (**C**) A number of BK(−) cells significantly increases in G2M phase comparing to G0/G1 and S. A percentage of stained (positive) and unstained (negative) cells was averaged from a minimum of 20 confocal images (6–10 сells per image) for each experimental condition. *Two means are significantly different in group and compared to synchronized (28 h) cells and 18 h timepoint, p < 0.05. ^#^No. S.D. inside and between the groups.

**Figure 4:**
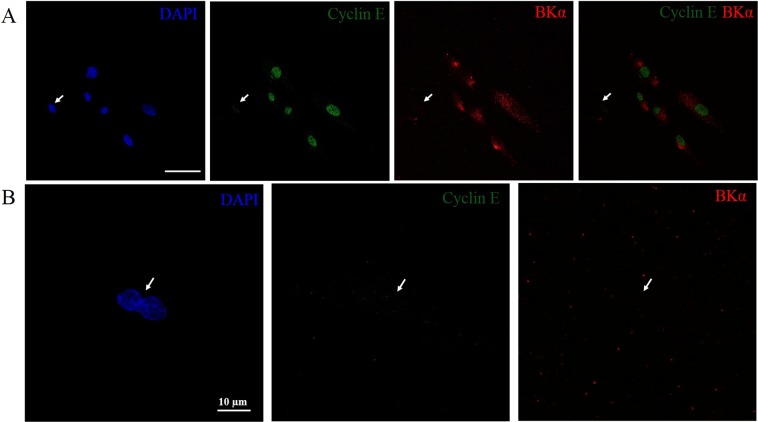
Co-immunostaining of eMSCs for BK channels and G1 phase marker Cyclin E. (**A**) Shown are representative images demonstrating that BK-negative cells are also Cyclin E negative. Scale bar 50 µm. (**B**) eMSCs having mitotic morphology (dividing cells) are also BK- and Cyclin E negative.

To test the hypothesis of cell cycle dependent expression of BK channels in eMSCs we firstly synchronized cell population in G0/G1 phase of cell cycle using serum starvation for 28 h. The synchronization of the cells was confirmed by cell cycle analysis using flow cytometry. Exposure of the cells to serum-free media resulted in about of 80% of cells stopped in G0/G1 stage (Fig. 3). Based on previously published data on cell cycle dynamics of eMSCs^11,12^, we analyzed the cells at 18 h and 22 h after activation of the cell proliferation by serum addition to achieve maximum number of the cells accumulated in S and G2M phase, respectively. We carried out parallel sets of experiments using flow cytometry of the cells and immunofluorescent staining of BK channels to reveal the possible change of BK channel expression during cell cycle progression. Cell cycle distributions of eMSCs at 18 and 22 h time points detected by flow cytometry are demonstrated on Fig. 3A. Importantly, immunofluorescent staining allowed us to reveal that the number of BK-negative cells significantly increases in G2M phase comparing to G0/G1 and S phases of the cycle (Fig. 3B,C). To confirm our results, cells were double stained for BK channels and Cyclin E as G1 phase marker. We showed that BK-positive cells are also positively stained for Cyclin E, whereas BK-negative cells are not stained for Cyclin E (Fig. 4A). Moreover, the analysis of the cell shape revealed, that the cells having mitotic morphology (dividing cells) are both BK- and Cyclin E negative that is in line with our observations (Fig. 4B).

### Selective BK channel pore blockers did not prevent cell cycle transitions of eMSCs

**Figure 5:**
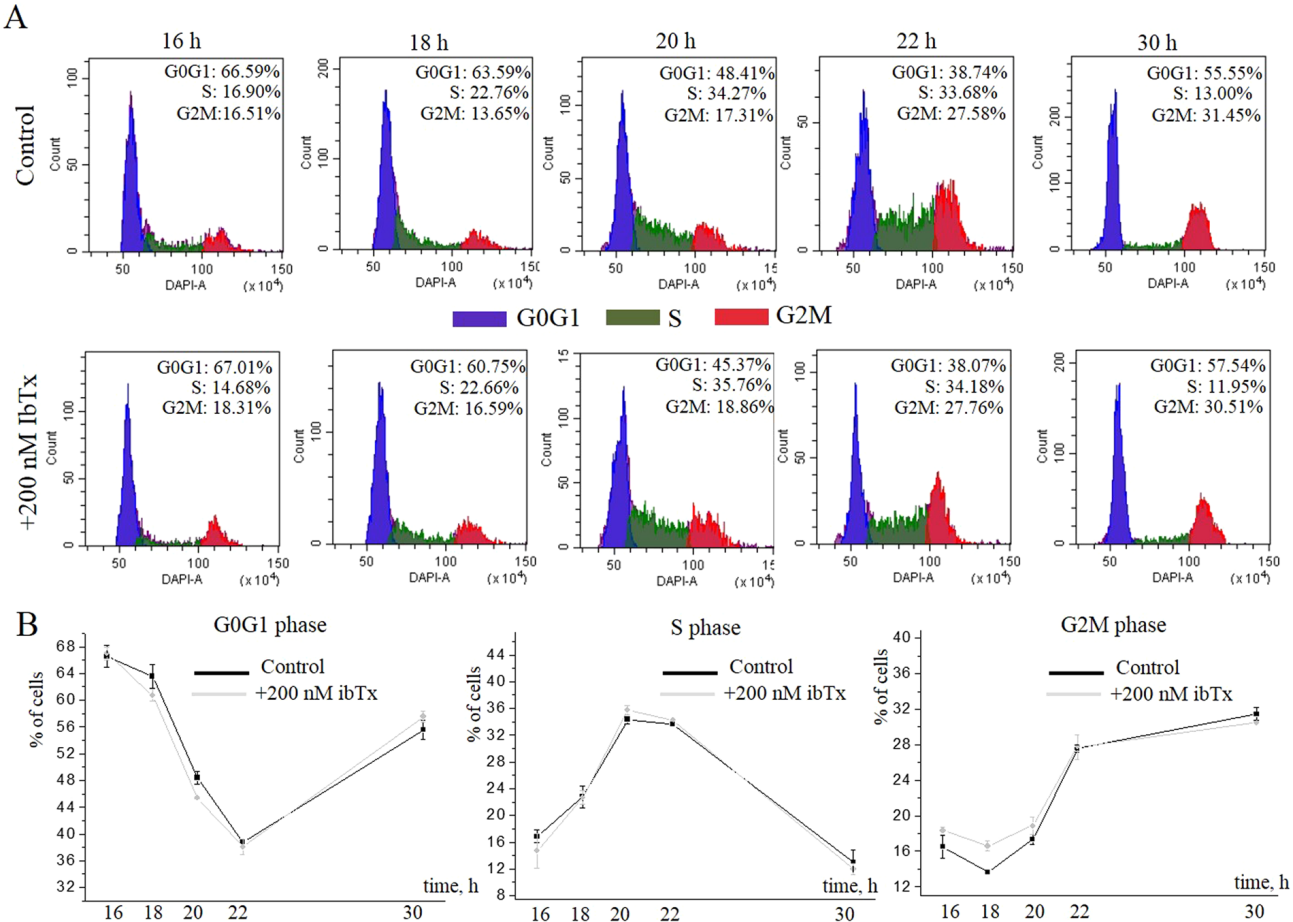
Cell cycle analysis of eMSCs in the presence of selective BK channel blocker iberiotoxin (IbTx). (**A**,**B**) Cell cycle distribution (**A**) and phase dynamics (**B**) at different time points in the control cells and in the sample with 200 nM IbTx.

We hypothesized that modulation of BK channel activity could significantly affect the transitions between different stages of eMSCs cycle. To test our hypothesis we analyzed cell cycle of eMSCs in the presence of selective BK channel inhibitors iberiotoxin (ibTx, 100–200 nM) or charybdotoxin (chTx, 100–200 nM)^13^. IbTx or chTx were added to the cells in full media after cell culture synchronization together with the induction of cell proliferation. Then, cell cycle of eMSCs in the presence or absence of the inhibitors was analyzed by flow cytometry 16, 18, 20, 22, 24 and 30 h after stimulation of the proliferation. A 30-hour point was taken to check the effect of toxins on the cell transition from the G2M phase to the next G0/G1 phase. Firstly, the potential effect of BK channel blockers on cellular viability was specifically addressed in our experiments. Using propidium iodide staining followed by flow cytometry analysis, it was found that neither iberotoxin nor charybdotoxin had any effect on cell viability (88.8% of viable cells comparing to 89.0% in control). Figure 5 shows the results of representative flow cytometry experiment demonstrating the percentage of cells in different phases of the cell cycle under the control condition (no toxin addition) and in the presence of ibTx in culture media. No change in the cell cycle distribution (Fig. 5A) or in the transition dynamics between different phases (Fig. 5B) is observed in the presence of selective inhibitor of BK channels. Consistently, we observed no effect of the other BK channel blocker, chTx, on cell cycle of eMSCs (Supplementary Fig. 4).

**Figure 6:**
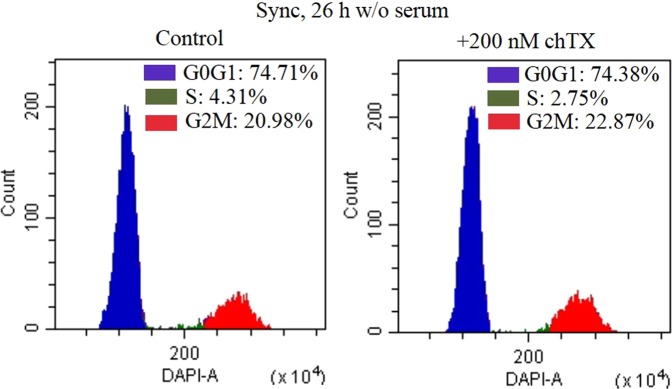
Cell cycle distribution of eMSCs after synchronization of the cells in serum free media in the control sample and in the presence of 200 nM of charybdotoxin (chTx). No effect of BK channel blocker chTX on eMSCs synchronization induced by serum starvation is observed. chTx was added directly to serum-free media and the cells were incubated in this medium for 26 h.

We further tested how the presence of the BK channel blockers affect cell synchronization by serum starvation. The results of our experiments clearly demonstrated, that synchronization (Fig. 6) and cell viability (data not shown) of eMSCs induced by incubation in serum free media for 26 h in the presence of chTx is not significantly different comparing to control cells.

Taken together, our data showed that both selective BK channel inhibitors did not significantly (p < 0.05) affect cells transitions between the phases of the cell cycle even at maximal concentration tested (200 nM). As a result, we conclude that the dynamics of the presence of BK channels on the membrane of eMSCs can be an indicator of cellular proliferation, but no contribution of BK channels as selective potassium-transporting pathway to the fundamental mechanism of cell passageway through the cycle is observed.

## Discussion

In current study we focused on the possible role of BK channels in cell cycle progression of eMSCs. We showed, that the fraction of unsynchronized cells were negatively stained with specific antibodies against pore-forming alpha BK subunit. The observed effect could not be explained by heterogeneity of cell culture, apoptosis or their differentiation status. Using cell synchronization we have shown that the presence of BK channel proteins on the plasma membrane is dependent on cell cycle stage and significantly decreases in G2M phase. The observed dynamics of the changes in the number of BK-positive cells during cell cycle progression is in principal agreement with “membrane potential hypothesis” of cell cycle progression and low permeability of plasma membrane of the cells for potassium ions in G2M phase. According to membrane potential hypothesis, changes in membrane potential control and regulate the transitions between cell cycle stages^6^. We proposed, that alteration of the expression of BK channels may reflect their putative contribution to cell cycle transitions of eMSCs. However, we demonstrated that the functioning of BK channels as selective potassium permeation pathway is not important for cycle progression as two selective pore blockers ibTx and chTx failed to affect the cell cycle of the cells.

The analysis of literature data showed that the effects of BK channel suppression on cell cycle reported earlier are rather controversial and could significantly differ between species, cell type and the origin of the cells. Particularly, in rat mesenchymal stem cells from bone marrow no effect of iberiotoxin or KCa1.1 siRNA on cell proliferation was observed^14^. At the same time, blocking of BK currents by paxilline or specific siRNA in human mesenchymal stem cells from bone marrow resulted in cell cycle arrest in G0/G1 phase^15^. In contrary, treatment of MCF-7 breast cancer cell line with ibTx or chTx for 4 days resulted in decrease of the number of the cells in G0/G1, increased the proportion of the cells in S and G2M stage, but failed to induce cell proliferation^16^. In neuroblastoma cells, inhibition of BK channels by TEA or ibTx induced cell proliferation via activation of staurosporine-sensitive protein kinases^17^ whereas no effect of ibTx on glioma cell proliferation was observed under normal culturing conditions^18^. In various breast cancer cells inhibition of BK channels resulted in increase, decrease or no effect on cell proliferation depending on particular cell line^19^.

It was shown that incubation of breast cancer cells with ibTx produced no effect on cell cycle whereas blocking of BK channels with ibTx significantly reduces the proliferation of breast cancer cells induced by extracellular ATP application followed by the increase of intracellular Ca^2+^ concentration^20^. Thus, it is reasonable to assume, that Ca^2+^ -activated BK channels could play some role in stimulus-induced cellular responses that are primary linked with the rise of internal Ca^2+^ concentration. Indeed, in our previous study we showed the BK channel activation by stretch-induced Ca^2+^ entry via mechanosensitive ion channels whereas no activity of BK currents was observed in physiological range of membrane potentials in the absence of mechanical stimuli^5^. The putative participation of BK channels in different stimulus-induced responses in eMSCs remains to be elucidated.

In several cell types the changes in the amount of KCNMA1 (Potassium Ca^2+^-activated Channel Subfamily M Alpha 1) mRNA was shown to be cell cycle dependent. Particularly, in rat mesenchymal stem cells from bone marrow the maximum and minimum amount of KCNMA1 mRNA was detected in early G1 and in the end of G1 phase of the cycle, respectively^15^. In contrary, the maximum of KCNMA1 was detected in the later G1 stage and decreased to S phase of the cycle in MCF-7 breast cancer cells^16^. However, it should be specifically noted, that the amount of mRNA may not reflect the changes in protein expression level during cell cycle. In our experiments, staining of eMSCs with selective antibodies against alpha-subunit of BK channel protein showed no significant change in the number of BK-negative or BK-positive during progression from G0/G1 to S phase whereas a number of BK-negative cells significantly increases in the cell population in G2M phase.

Our approach with the use of two selective pharmacological BK channel pore blockers is significantly relevant in probing the role of BK proteins as an ion conductive potassium pathway implicated in regulation of membrane potential in eMSCs during cell cycle transitions. A number of channel proteins, including voltage-gated potassium channels (K_v_), the close structural homologs of BK channels, were reported to have, additionally to their functioning as ion transporters, a number of non-conductive properties^21,22^. Although no information is available to date on non-channel function of BK channels, taking into account their structural proximity to canonical Kv channel proteins, one can hypothesize that alpha subunits of BK channels could also have some functions that are not related with their ion transporting role. Importantly, the formation of complexes or even direct physical co-association of BK subunits with several proteins including a number of other ion channels (i.e. NMDA receptors, Orai1, Ca_v_ or TRP) was previously demonstrated^10,23–25^. The formation of functional calcium-regulated complex between mechanosensitive and BK channels was reported in our previous study^5^. Thus, potential non-conductive role of BK channels in cell cycle progression could not be excluded. In this case, the use of gene-specific knockdown of BK channels (i.e. by siRNA) could not be a reliable tool to probe the role of BK channels as selective potassium transporters during cycle progression comparing to pharmacological channel pore blockers. Revealing of another molecular correlates, that control the potassium efflux during cell cycle progression of eMSCs could be a specific goal for the future studies. Indeed, in eMSC other types of KCa channels (IK, SK) were detected using patch-clamp technique^5^ and RT-PCR (unpublished data).

BK channels are important physiological regulators of cellular reactions in a number of highly specialized cells and tissues. In excitable cells of nervous system BK channels are critical regulators of firing potential, neuronal excitability, neurotransmitter release and neurovascular coupling^26^. The role of BK channels in non-excitable cells remains poorly understood. Generally, KCa channels are considered to play a significant role in Ca^2+^ signaling in excitable and non-excitable cells of different origin through their ability to be activated by the change in intracellular Ca^2+^ concentration^9^. The role of BK channels in stem cell physiology is rather controversial and seems to be dependent on the source and stem cell type. In eMSCs derived from bone marrow BK channel inhibition resulted in cell cycle arrest in G0/G1 stage, reduced adipogenic and decreased osteogenic differentiation^15^. In contrast, no changes in cell cycle were demonstrated in rat MSCs from bone marrow in response to BK channel block^14^. The results of our experiments demonstrate cell cycle dependent expression of BK channels in human MSCs derived from desquamated endometrium. We revealed in this study, that BK channels could be considered as a potential markers of eMSCs in different phases of the cell cycle. At the same time, pharmacological block of BK channels does not affect proliferative activity, viability or cell cycle transitions indicating their minor role as selective potassium transporting pathways during cycle progression in eMSCs.

## Materials and Methods

### Cell culture and reagents

The human endometrial mesenchymal stem cell (eMSCs) line was obtained and characterized in the Institute of Cytology RAS (St. Petersburg, Russia). The eMSCs were cultured according to a standard protocol in DMEM/F12 medium (Gibco, USA) supplemented with 10% bovine fetal serum (HyClone, USA), 1% antibiotic-antimycotic solution and 1% GlutaMAX (Gibco, USA). Cells were passed at a density of 1:3-1:4 twice a week, using 0.05% trypsin/EDTA (Invitrogen, USA)^11^. For patch-clamp and immunocytochemical experiments cells from 5 to 9 passages were seeded on 4 × 4 mm or 12 × 12 mm coverslips respectively. Iberiotoxin (ibTx) and charybdotoxin (chTx) were purchased from Tocris (USA). Stocks were diluted in distilled water, and the working solutions were prepared by adding a necessary amount of stock to the culture media.

### Electrophysiology

For measurement of single currents through BK channels cell-attached configuration of patch-clamp method was used. The patch clamp setup was based on AxoPatch 200B amplifier and Digidata 1550 A Analog-Digital Interface (Molecular Devices Corp., USA) controlled by pClamp 10.5 software. Pipettes of 7–10 mOhm resistance were made from borosilicate glass (BF 150-110-10 F, Sutter Instruments, USA) on P-97 puller (Sutter Instruments, USA). The extracellular solution (in the pipette) in cell-attached configuration contained (mM): 145 NaCl, 2 CaCl_2_, 1 MgCl_2_, 10 HEPES/TrisOH; the chamber was filled with (mM): 145 KCl, 2 CaCl_2_, 1 MgCl_2_, 10 HEPES/TrisOH to nullify resting membrane potential. Channel activity was recorded in the range of membrane potentials from +0 to +150 mV. Recordings were filtered, analyzed and processed in Clampfit 10.5 (Molecular Devices Corp., USA) and Qtiplot (Iondev SRL., Romania). The activity of BK channels was estimated by:(1) number of channel openings on different membrane potentials, 50% of open value of the current was considered as an opening; (2) as open channel probability (NP_o_) using following equation: NP_o_ = I/i, where I is a mean current determined from the amplitude histograms, i is the unitary current amplitude.

### Immunocytochemistry

For immunofluorescent staining, cell fixation was performed in 3.7% paraformaldehyde for 10 min at room temperature (RT), then the cells were washed 3 times with phosphate buffered saline PBS. Nonspecific binding of the antibodies was blocked by incubating the samples in PBS containing 10% serum for 1 hour at RT. For detection of BK channels in plasma membrane of eMSCs cells were incubated with relevant primary Anti-KCa1.1 (anti-BK alpha subunit) antibodies against the extracellular epitope of the targeted protein (APC-151, Alomone Labs, Israel) for 20–24 hours at +4 °C. Staining with fluorescently labelled secondary antibodies (Goat anti-rabbit conjugated to Cy3, Santa Cruz, USA) was performed for 1 hour at RT in the dark. No staining was observed after pre-incubation of the primary antibodies with control blocking peptide (BP, 1 h, Supplementary Fig. S1) or after exposure of the cells only to secondary fluorescent antibodies (data not shown). For double co-staining of G1 phase marker Cyclin E and BK channels, cells were fixed with 3.7% paraformaldehyde for 10 min at (RT), washed 3 times with PBS, blocked with 10% goat serum (1 h, RT) and incubated for 1 h with anti-BK antibodies followed by secondary GAR-Cy3 (1 h, RT). Then, the cells were permeabilized with 0.1% Triton TX-100 (10 min at RT), blocked with 10% goat serum (1 h, RT) and incubated with anti-Cyclin E primary antibodies (Santa Cruz, USA) for 20–24 hours at +4 °C followed by Alexa488-Goat anti mouse (Alexa488-GAM, Invitrogen, USA) secondary antibodies incubation for 1 h at RT. Cell nuclei were counterstained with of 4′,6-diamidino-2-phenylindole (DAPI, 0.05 μg/ml, 30 min at RT). Visualization of the fluorescently labeled preparations were performed on Leica TCS SP5 (Leica Microsystems GmBH, Germany) or Olympus FV3000 (Olympus, Japan) confocal microscopes equipped with appropriate combinations of lasers and detectors. 40x oil objectives were used for image acquisition. Images were analyzed in ImageJ (NIH, USA) software. A mean percent of stained and unstained cells was calculated and averaged from at least 20 confocal images (6–10 сells per image) on each timepoint. Mean numbers of BK-negative and BK-positive cells were compared using paired Student’s t-test (p < 0.05 was considered as significant): (1) - inside each timepoint and (2) between the taken timepoints.

#### CD cell marker phenotyping

Since differentiation status of stem cells is tightly connected to cell cycle, we analyzed the eMSCs population for the expression of the main surface markers of human mesenchymal stem cells. For immunophenotypic analysis of CD markers a single cell suspension was obtained using 0.05% trypsin and EDTA (Invitrogen, USA). Cells (1 × 10^6^/ml) were resuspended in PBS solution containing 5% fetal bovine serum. The cells were treated with FITC or phycoerythrin-labeled antibodies to CD34, CD44, CD105, HLA-DR (Beckman Coulter), CD73 (BD Biosciences) and CD90 (Chemicon) and assayed by flow cytometry. Cell fluorescence was measured using CytoFLEX (Beckman Coulter, USA) flow cytometer. CD-phenotyping of cells was conducted in parallel with other experiments described in this article. We found that all cells of the analyzed population express CD 73, CD 90, and CD 105; and do not express CD 34 and HLA-DR (Supplementary Fig. S2). These results are in accordance with the minimal criteria for defining multipotent mesenchymal stromal cells stated by the International Society for Cellular Therapy^27^. According to the expression profile of surface CD markers, the analyzed population is homogeneous. It is important to note that 100% of the analyzed cells express CD90 marker of stemness, i.e. the cells used in our study have the status of stem, non-differentiated cells.

### Synchronization of the cells and cell cycle analysis

eMSCs were synchronized in the G0 phase of the cell cycle by serum starvation for 28 h. The stimulation of the cell proliferation was performed by serum addition. After initiation of cell proliferation, cell cycle phase distribution was analyzed at different time points. The adherent cells were rinsed with PBS, harvested using trypsin-EDTA solution and suspended in the growth medium. Cells were permeabilized with 0.1% Triton X-100 (Sigma) and stained for 5 min with 2 μg/ml DAPI. Cell cycle phase distribution was measured with CytoFLEX flow cytometer (Beckman Coulter; 405 nm laser) and analyzed using CytExpert 2.0 software. Fluorescence from 10,000 cells was collected.

### Cell viability assay

To estimate the potential cytotoxic effect of BK channel pore blockers IbTx and ChTx cell viability assay was performed. The percentage of dead and living cells was estimated by flow cytometry using propidium iodide staining. Cells were harvested with 0.05% trypsin-EDTA solution, suspended in the growth medium, stained with 50 μg/ml propidium iodide for 5 min and analyzed with CytoFLEX flow cytometer (Beckman Coulter; 488 nm laser). Cells were gated by size and granularity on FSC/SSC plot and cell debris was excluded from the analysis. Mean fluorescence intensity from 10,000 cells was acquired.

### Statistics

Data are presented as mean ± SEM (*n* – number of experiments) and were compared using standard paired Student’s *t*-test, p < 0.05 was considered significant.

## Supplementary information

Supplementary Material
