# Supplementary material for: Cell Cycle-Dependent Expression of Bk Channels in Human Mesenchymal Endometrial Stem Cells
Source: Sci Rep. 2019 Mar 14;9:4595. doi: 10.1038/s41598-019-41096-2 (PMC6418245; doi:10.1038/s41598-019-41096-2)
Supplement: Supplementary file 1 — Supplementary Material [file 41598_2019_41096_MOESM1_ESM.pdf]

CELL CYCLE-DEPENDENT EXPRESSION OF BK CHANNELS IN HUMAN MESENCHYMAL ENDOMETRIAL STEM CELLS

Vladislav I. Chubinskiy-Nadezhdin[1*], Anastasia V. Sudarikova[1], Mariia A. Shilina[1], Valeria Y. Vasileva[1], Tatiana M. Grinchuk[1], Olga. G Lyublinskaya[1], Nikolai N. Nikolsky[1], Yuri A. Negulyaev[1,2]

1 Institute of Cytology RAS, 194064 Tikhoretsky Ave. 4, St. Petersburg, Russia,

2 Department of Medical Physics, Peter the Great St. Petersburg Polytechnic University, 29, Polytechnicheskaya st., 195251, St. Petersburg, Russia

***Corresponding author:** Vladislav I. Chubinskiy-Nadezhdin, Institute of Cytology RAS, 194064, Tikhoretsky Ave. 4, St. Petersburg, Russia

Tel: +7-812-297-14-97; Fax: +7-812-297-35-41.

E-mail address: vchubinskiy@gmail.com

Figure S1.

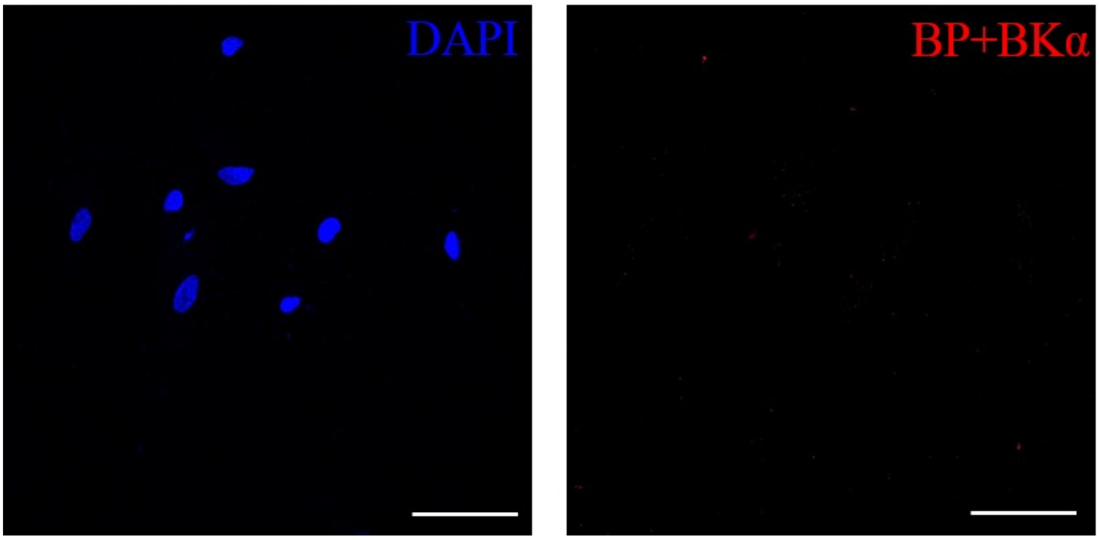

Figure S2

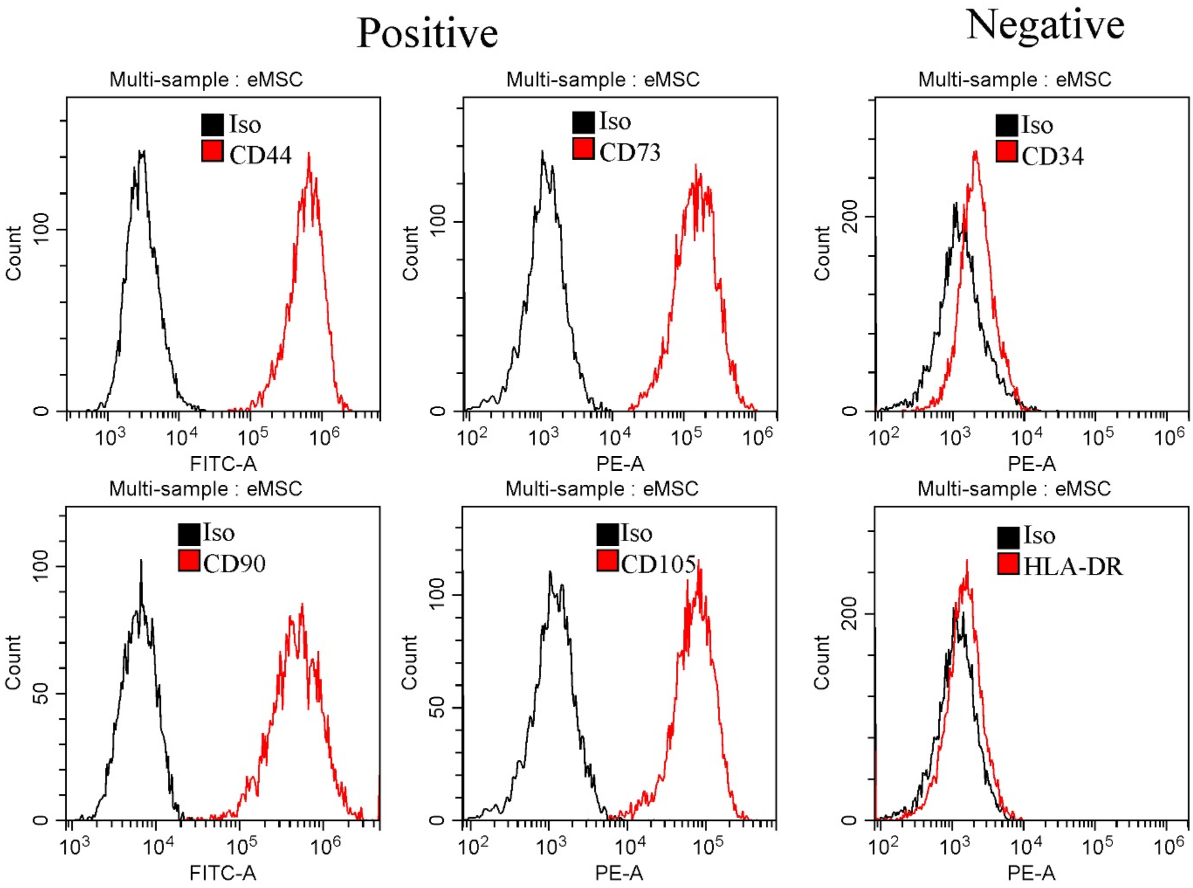

Figure S3.

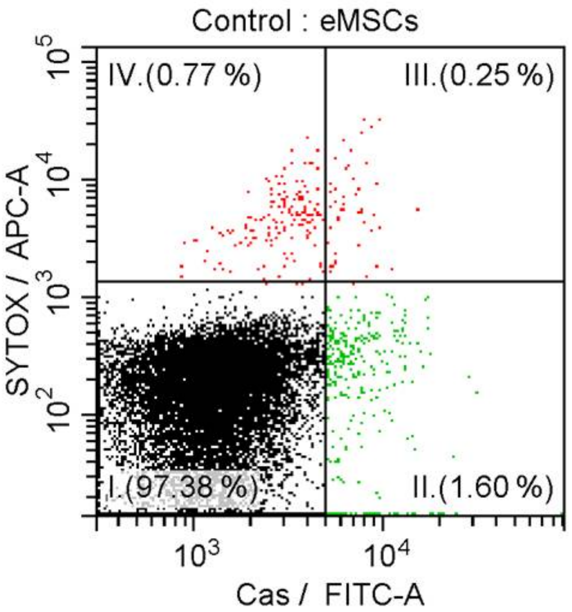

Figure S4

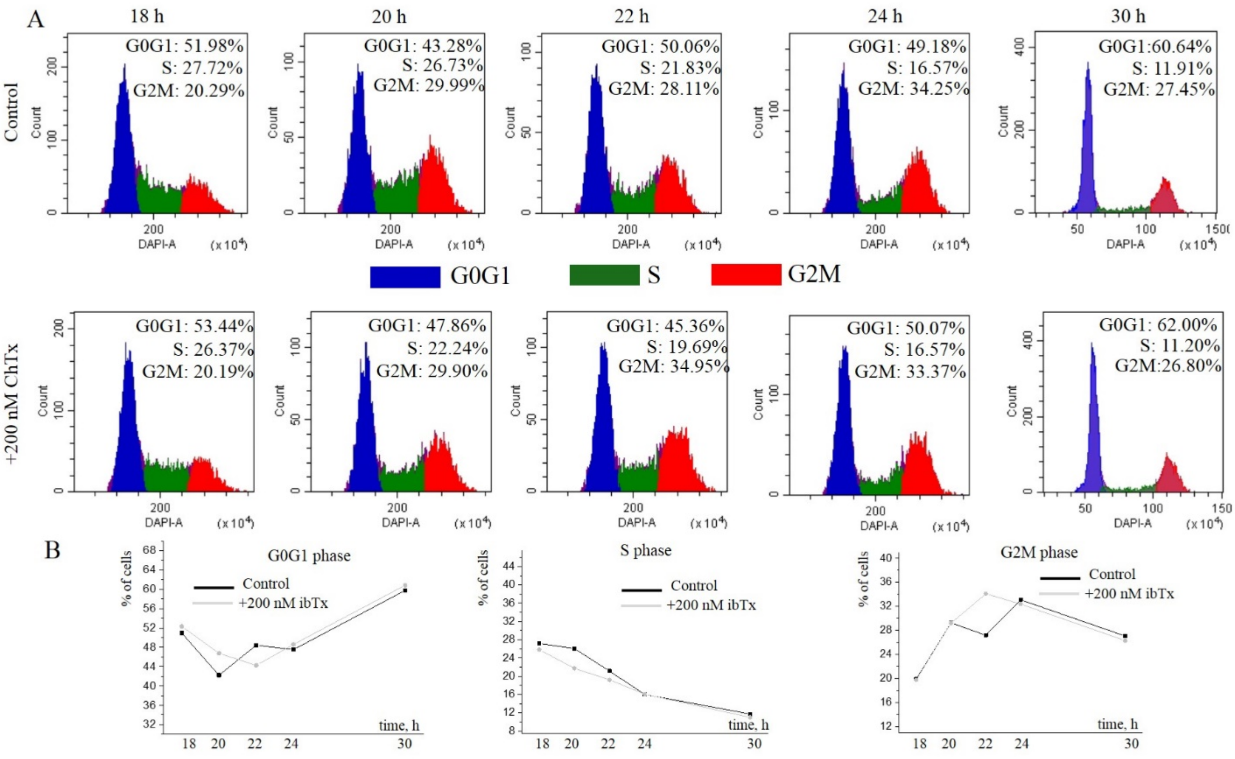

Supplementary Figure Captions:

Figure S1. The specificity of BK channel staining was confirmed by pre-incubation of primary anti-BK antibodies with specific blocking peptide (BP). No staining is observed after 1h of incubation with BP. Scale bar 50 μm.

Figure S2. Flow cytometry analysis revealed the positive expression of CD73, CD105, CD90 and CD44, as well as negative expression of CD34 and HLA-DR surface markers in eMSC cultures (in accordance with the minimal criteria for defining multipotent mesenchymal stromal cells stated by the International Society for Cellular Therapy[27]. Red line – CD markers, black line – isotype controls (IgG).

Figure S3. eMSCs maintain low basal level of apoptosis. Prior to the flow cytometry analysis, cells were stained with CellEvent™ Caspase-3/7 Green Detection Reagent (ThermoFischer Scientific, Waltham, USA) according to manufacturer's protocol. Positive control: cells after 24 h incubation with 200 μM of H_2O_2.

Figure S4. chTx does not interfere with cell cycle progression of eMSCs. Shown are representative results of the experiment demonstrating cell cycle distributions (A) and dynamics of the transitions between phases (B) in control cells and in the presence of 200 nM of chTx.
